# Effects of essential oils on foulbrood bacteria and honey bee workers (*Apis mellifera*) under laboratory conditions

**DOI:** 10.3389/finsc.2026.1787016

**Published:** 2026-04-01

**Authors:** Cinzia Marianelli, Laura Narciso

**Affiliations:** Department of Food Safety, Nutrition and Veterinary Public Health, Istituto Superiore di Sanità, Rome, Italy

**Keywords:** *Apis mellifera*, broth microdilution assay, essential oils, spot-on-agar test, toxicity bioassay

## Abstract

**Introduction:**

American and European foulbrood diseases (AFB and EFB, respectively) result in considerable economic losses for beekeepers. Currently, no satisfactory methods are available for the treatment of either disease. There has been a recent surge of interest in the use of natural substances, such as essential oils (EOs), as a means of combating infections in apiaries. The objective of this study was to evaluate the *in vitro* antimicrobial activity of a number of EOs against the causative agents of AFB and EFB, namely *Paenibacillus larvae* and *Melissococcus plutonius*, respectively, and their safety towards bees.

**Methods:**

The antimicrobial activity of 18 EOs was initially assessed *in vitro* against the two foulbrood bacteria by the spot-on-agar test, where the EOs were deposited directly onto the agar surface. The EOs demonstrating the most significant antimicrobial activity against one or both of the bacterial pathogens were then selected for further assessment of their toxicity towards the foulbrood bacteria by the resuzurin-based microdilution method and towards adult bees using the vapor exposure bioassay at one and three hours.

**Results:**

The study revealed that oregano, juniper, sage, thyme, cinnamon, cumin, clove and black pepper were the most effective against the foulbrood bacteria. The results of the exposure bioassays demonstrated that bees exposed to those EOs exhibited greater tolerance to the vapor of cinnamon and oregano after one hour of exposure than the other EOs. As the duration of exposure increased to a period of three hours, an escalation in toxicity was observed.

**Discussion:**

The preliminary findings of the present study suggest EOs as potential candidates for the development of new natural antimicrobial treatments against foulbrood diseases. However, further *in vitro* larval bioassays and field trials are needed to validate their clinical effectiveness and safety.

## 1 Introduction

American and European foulbrood diseases (AFB and EFB, respectively) cause significant economic losses to beekeepers (1). Both diseases affect honey bee larvae and not adult bees. However, adult bees may facilitate the spread of infection within a colony (2).

AFB is caused by *Paenibacillus larvae*, a Gram-positive, spore-forming rod bacterium. Bacterial spores germinate in the gut of larvae, multiply, and kill the larvae at the pre-pupal or pupal stage (3). AFB is one of the most contagious and destructive diseases, capable of causing the collapse of the entire colony (4). The prevention of disease in bees is contingent upon the implementation of good beekeeping practices and the maintenance of robust, healthy colonies. Conversely, disease control proves challenging for beekeepers and researchers due to the resilience of spores to unfavourable conditions and their extended periods of viability (5).

EFB is caused by *Melissococcus plutonius*, a Gram-positive bacterium and is closely related to AFB in symptomatology. The pathogen infects unsealed larvae and kills them at the age of 4 to 5 days. The larvae then undergo a process of decomposition, turning yellowish, then brown, before becoming watery (6). EFB has a worldwide distribution and is considered to be less dangerous than AFB. However, the prevalence and severity of EFB appear to be influenced by environmental factors, including climatic conditions (7).

Currently, no satisfactory methods are available for the treatment of either AFB or EFB. Although the use of antibiotics in beekeeping is prohibited in the European Union, where the burning of AFB or EFB affected combs is the norm, it is permitted in the United States, South America, and some East Asian countries (8). Tetracycline-resistant strains of *P. larvae* have been identified in the USA, Canada, and Argentina (9–11). Furthermore, antibiotic residues can persist in honey and reduce its quality for human consumption. Thus, the development of novel therapeutic strategies for the treatment of honey bee colonies infected with either AFB or EFB is imperative.

In recent decades, there has been a growing interest in the use of natural substances, such as essential oils (EOs), to fight infections in apiaries (12, 13). EOs are mixtures of terpenes, terpenoids and phenolic compounds and are known to have strong antimicrobial activity (14, 15). Moreover, they do not develop bacterial resistance or adaptation, are eco-friendly and, in general, safe for honey bees and beekeepers (14). No adverse effects have been observed in adult bee workers following topical or oral administration of EOs (16). In fact, EOs have been demonstrated to enhance the general health of bees (17), survival (18) and immune gene expression (18, 19). A number of studies have demonstrated the antimicrobial efficacy of several EOs against *P. larvae*, in both laboratory-based (17, 20–22) and in field trials (17, 23, 24). However, there are fewer records on the antimicrobial activity of EOs against *M. plutonius* (24–26).

To date, no universally accepted standardized protocol has been established specifically for the assessment of the antimicrobial properties of EOs, due to their volatility and peculiar physicochemical properties. A number of *in vitro* testing methodologies, comprising both qualitative (or semi-quantitative) and quantitative approaches, have been documented in the extant literature (27). Qualitative methods include agar diffusion techniques, which use paper disks impregnated with an EO and positioned on the agar surface (27, 28), or wells (holes) drilled out into the agar and filled with an EO (27). Alternatively, the EOs are deposited directly onto the agar surface in a process referred to as the spot-on-agar test (29–31). The agar diffusion technique is a common method employed for the preliminary screening of the antimicrobial activities of EOs. This technique is based on the measurement of the size of the zone of inhibition (27, 32). Quantitative methods include broth dilution (where an EO is diluted at varying concentrations in a liquid medium) (27, 28, 32, 33), and agar dilution (where an EO is mixed at varying concentrations with melted agar) (27), and these methods are used for the determination of Minimum Inhibitory Concentrations (MICs). Despite the fact that antimicrobial effects of EOs have undergone extensive *in vitro* research for many decades, the existing literature on EOs is characterised by considerable heterogeneity in terms of the methods employed, the growth media utilised, and the EOs examined. Consequently, the comparison of results is rendered more challenging. It is evident that further research is required in order to facilitate the establishment of a universally accepted methodology for the assessment of the antimicrobial properties of EOs and their compounds.

The objective of this study was to evaluate the antimicrobial activity of 18 EOs against the causative agents of foulbrood diseases, specifically *M. plutonius* and *P. larvae*. Additionally, the toxicological effects caused by the fumigant activity of those EOs exhibiting greater efficacy against the foulbrood bacteria in adult honey bees (*Apis mellifera*) were evaluated under laboratory conditions, in order to gather data for the development of new natural antimicrobial treatments against both AFB and EFB.

## 2 Materials and methods

### 2.1 Essential oils

The study included 18 EOs, which were produced through steam distillation and supplied by Cruciani Prodotti Crual Srl (Rome, Italy): cinnamon (*Cinnamomum zeylanicum*), bergamot (*Citrus bergamia*), lemon (*Citrus limonum*), cumin (*Cuminum cyminum*), juniper (*Juniperus communis*), lavender (*Lavandula angustifolia*), laurel (*Laurus nobilis*), tea tree (*Melaleuca alternifoglia*), peppermint (*Mentha piperita*), myrtle (*Myrtus communis*), basil (*Ocimum basilicum*), oregano (*Origanum vulgare*), black pepper (*Piper nigrum*), rosemary (*Rosmarinus officinalis*), sage (*Salvia sclarea*), clove (*Syzygium aromaticum*), thyme (*Thymus vulgaris*) and ginger (*Zingiber officinale*).

### 2.2 Foulbrood bacteria and spot-on-agar test

The reference strains *P. larvae* (CCM 4484, other number LGM 16150) and *M. plutonius* (CCM 3707, other number ATCC 35311), provided by the Czech Collection of Microorganisms of the Masaryk University in Brno, Czech Republic, were used in the study. The two strains were cultured following the producer’s instructions: *P. larvae* at 37 °C in the medium B98 (10.0 g Mueller-Hinton, 15.0 g yeast extract, 2.0 g glucose, 3.0 g K_2_HPO_4_ and 1.0 g sodium pyruvate per litre); *M. plutonius* at 30 °C in anaerobic condition in the medium B64 (2.5 g yeast extract, 2.5 g peptone, 5.0 g neopeptone, 2.0 g tryptone, 2.0 g starch, 10.0 g glucose, 50 mL KH_2_PO_4–_1 mol/L pH 6.6 and 2.5 mL L-Cysteine-HCl 10% per litre).

The antimicrobial activity of the EOs were tested against both *P. larvae* and *M. plutonius* by the spot-on-agar test to minimize potential interference from disk material fibres or uneven well-edge diffusion. Petri dishes with a diameter of 90 mm and filled with approximately 20 mL of agar medium were employed. *P. larvae* and *M. plutonius* are both fastidious and slow-growing bacteria and exhibit poor growth under laboratory conditions. The two strains of foulbrood bacteria were cultured in their respective media and under their respective culture conditions for a period of 4 days (*P. larvae*) or 6 days (*M. plutonius*) prior to conducting the test, in order to achieve a turbidity of approximately 0.5 McFarland. In the absence of standardised procedures for the testing of EO properties, the approach adopted was to follow those for antimicrobial susceptibility testing, where the inoculum typically matches the turbidity of a 0.5 McFarland standard. Then, 500 μL of the *P. larvae* or *M. plutonius* suspension were spread onto the surface of each B98-agar or B64-agar plate, respectively. After the inoculum was absorbed into the agar, one microlitre of EO was applied on the agar surface, as previously described (31). On the same plate, one microlitre of PBS was added as a control. The plates were then incubated either aerobically at 37 °C for 4 days for *P. larvae* or anaerobically at 30 °C for 6 days for *M. plutonius*. Each experiment was conducted in triplicate on two separate occasions. The diameter of each zone of inhibition or halo was then measured in centimetres using a calliper and the mean values and standard deviations were subsequently calculated. According to a recent study of our group (31), the antimicrobial activity of EOs was considered very high when the inhibition halo diameter (hd) was greater than or equal to 2.0 cm (hd ≥2.0), high when hd was between 1.5 and 2.0 cm (1.5≤ hd <2.0), moderate when hd was between 1.0 and 1.5 cm (1.0 ≤ hd <1.5), low when hd was between 0.8 and 1.0 cm (0.8 ≤ hd <1.0), very low when hd was between 0.8 and 0.5 cm (0.5 ≤ hd < 0.8), and null when hd was less than 0.5 (hd < 0.5). The most effective EOs in combating the foulbrood bacteria were identified (see Results) and further investigated by the broth microdilution method to ascertain the MIC values. Concurrently, the selected EOs were tested for toxicity to adult bee workers by vapour-only exposure (or fumigation) (see section 2.4 for details). The main characteristics of the selected EOs are reported in **Table 1**. These characteristics were not determined by gas chromatography-mass spectrometry (GC-MS) measurements, but rather derived from the manufacturer’s reports (if provided) or the extant literature.

**Table 1:** Characteristics of the most effective EOs against the foulbrood bacteria.

EO	Plant name (plant source)^	Main components*
Cinnamon	*Cinnamomum zeylanicum* (leaf)	cinnamic aldehyde (65-80%), eugenol (4-10%) °
Cumin	*Cuminum cyminum* (seed)	camphene (25-30%), cuminic aldehyde (20-30%) °
Juniper	*Juniperus communis* (berry)	α-pinene (27-35%), sabinene (10%), limonene (2-7%) (34)
Oregano	*Origanum vulgare* (leaf and flower)	carvacrol (80-85%), *γ-*terpinene (5-7%), *p*-cymene and thymol (30)
Black pepper	*Piper nigrum* (berry)	monoterpene (70-80%) °
Sage	*Salvia sclarea* (flower)	linalyl acetate (45-70%) °
Clove	*Syzygium aromaticum* (bud)	eugenol (85-95%) °
Thyme	*Thymus vulgaris* (leaf and flower)	thymol and carvacrol (60%) °

^The part of the plant used to produce the EO is indicated in brackets, as specified by the manufacturer. * The chemical composition was obtained from either the manufacturer (°) or relevant literature in the case of missing data (the relevant reference is provided). All the EOs used in this study were produced through steam distillation of the respective plant parts.

### 2.3 Determination of minimum inhibitory concentration

The most efficacious EOs against the *P. larvae* and those most effective against *M. plutonius* (see Results) were then subjected to further investigation by the broth microdilution method.

The experiment was conducted in quadruplicate in 96-well plates. Media B98 and B64, with 0.1% Tween 80 to create a stable emulsion, were used for the testing of *P. larvae* and *M. plutonius*, respectively. A volume of 100 µL of the medium was inoculated into each well. Subsequently, EOs were added, and two-fold serial dilutions ranging from 10 to 0.019 μL/mL were conducted. Then, 100 µL of a bacterial suspension containing approximately 1.5 x 10^6^ CFU/mL of a foulbrood pathogen was inoculated into each well. The broth supplemented with Tween 80 was also used to set up a growth control containing no EO, as well as a sterile control without bacteria. Plates were then covered with EVA push caps (Micronic, The Netherlands) and incubated at 37 °C for four days (*P. larvae*) or at 30 °C for seven days (*M. plutonius*). Finally, 30 µL of freshly prepared 0.01% resazurin solution (Acros Organics, USA) was added to each well. The plates were then incubated overnight at 37 °C and subsequently assessed for the presence of chromatic alterations. A shift from blue to pink indicated a reduction of resazurin and therefore bacterial growth. The MIC values were defined as the lowest concentration of the tested EO that prevented a change in the color of resazurin from blue to pink.

### 2.4 Honey bee workers and vapor-only exposure bioassay

The exposure bioassay was performed in order to identify the EOs that demonstrated efficacy in combating the foulbrood bacteria without compromising bee survival.

**Figure 1:**
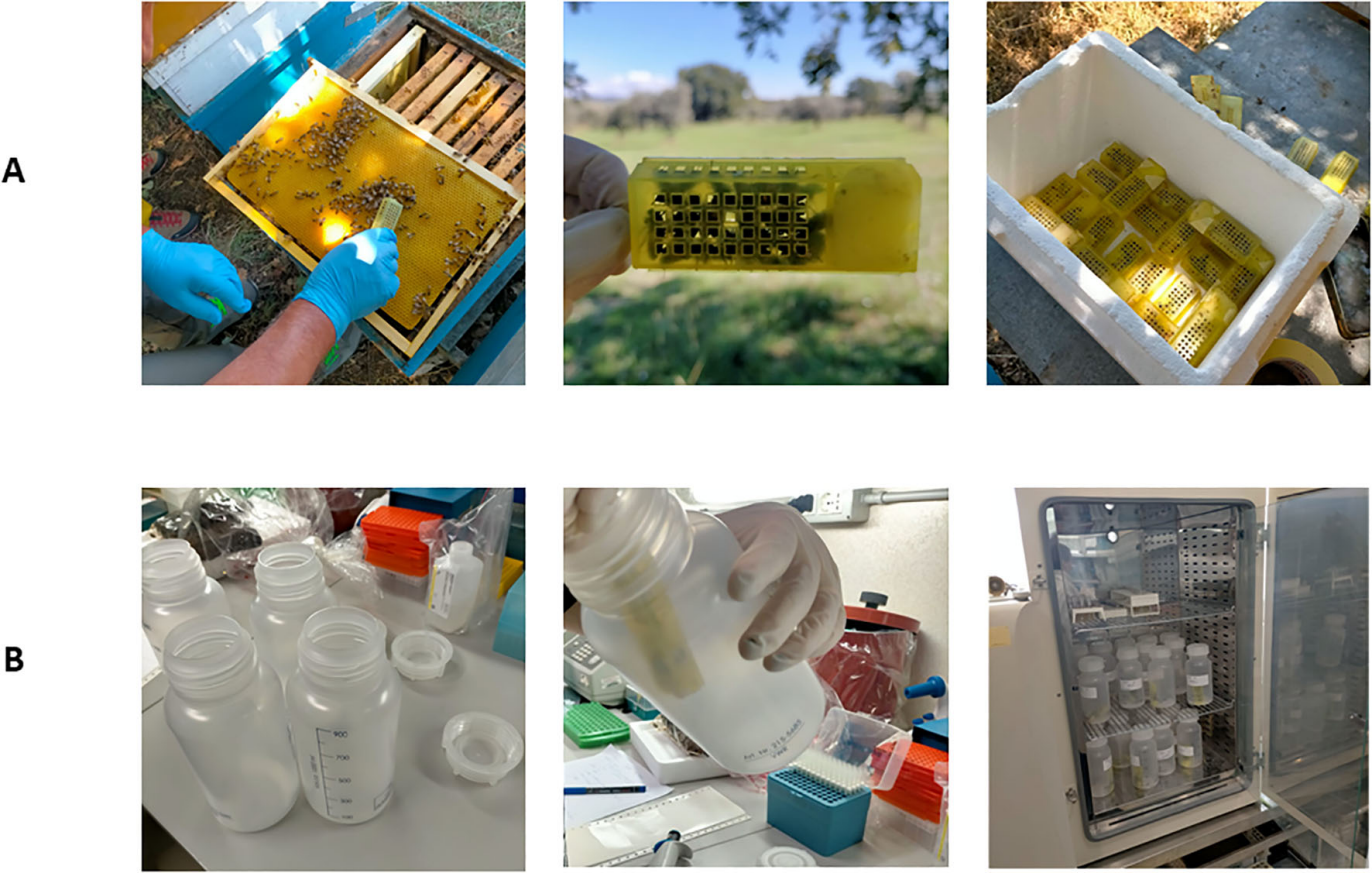
Honey bee worker collection and exposure bioassay. Panel **(A)** shows how the bees were collected from frames by brushing them into a queen bee cage, which had previously been filled with a white candy plug in the candy section. Panel **(B)** shows the exposure bioassays, which were conducted in 1 L polypropylene bottles containing a filter paper disc impregnated with an essential oil (EO) and two bee-filled cages. The bottles were then closed and incubated at 28 °C for 20 hours.

Honey bee workers from healthy colonies of *Apis mellifera* were sampled in an apiary located in the district of Alberese (Grosseto, Italy) on a summer day. The bees were collected from frames by brushing them into a 3.7 x 8.0 cm queen bee cages that were previously filled with a white candy plug in the candy section (about 7–10 bees per cage) (**Figure 1A**). The cages filled with bees were transported to the laboratories of Istituto Superiore di Sanità. The toxicity of EOs to adult honey bees following vapour-only exposure to EO-embedded paper was investigated.

Exposure bioassays were conducted in 1-L polypropylene bottles (VWR International, LLC) in order to investigate the toxicological potential of volatile plant-derived compounds of EOs towards honey bees. Filter paper discs with a diameter of 8 cm were embedded with 0.7 mL of sterile water and then impregnated with 50 µL of a single pure EO each. Two cages filled with bees was then inserted vertically per bottle (approximately a total of 15–20 bees per bottle) and placed against the body to avoid any contact between the bees and the EO-impregnated disc. The cap was then tightly screwed on to prevent loss of active compounds. Controls consisted of bottles containing discs embedded with water only (no EO) and cages with bees, were also prepared. All bottles were incubated at 28 °C for 20 hours (**Figure 1B**).

Essential oil vapours at a concentration of 50 µL/L air were generated inside each closed bottle. The vitality of the insects was observed after one hour and three hours of exposure, as well as after 20 hours. Bees that were able to move freely in the cage were considered alive. Bees that showed isolated movements of one or more legs were recorded as inactive. Completely immobile bees were classified as dead. Bee mortality was assessed by checking for the total absence of movement and the lack of response to physical stimulation at the conclusion of the experiment. The neutralization rate of each EO was determined by dividing the total number of inactive and dead insects by the sample size, according to Castagna et al. (35). The experiment was repeated on two separate occasions. The weighted mean neutralization rates for the two independent experiments and the standard deviations were then calculated.

### 2.5 Statistical analysis

The efficacy of EOs against *P. larvae* and *M. plutonius* was evaluated by analysing the extent of bacterial growth inhibition through the measurement of the inhibition halo diameters (hds) after four (*P. larvae*) or six (*M. plutonius*) days of incubation. The Shapiro-Wilk test was used to assess data distribution. Mean values of hds and standard deviations (SDs) were used. Two-way ANOVA followed by Sidak’s multiple comparisons test was performed to determine the significance of the main effects of EO exposure (E, 18 EOs) and foulbrood bacterial pathogen (P, *P. larvae* and *M. plutonius*), as well as the interaction between them (E x P).

The toxicological effect of the 18 EOs at two distinct time points – one and three hours of exposure - was evaluated by the weighted mean neutralization rates and standard deviations (SDs). Two-way ANOVA followed by Sidak’s multiple comparisons test were performed to determine the significance of the main effects of EO exposure (E) and exposure time (T), as well as of the interaction between them (E x T).

The statistical analyses were conducted using GraphPad Prism 9 version 9.0.0 for Windows, GraphPad Software, La Jolla, California, USA (www.graphpad.com). Differences were considered significant at a *p*-value of less than 0.05. (*p* < 0.05).

## 3 Results

### 3.1 Antimicrobial activity of EOs against foulbrood bacteria: spot-on-agar tests

**Figure 2:**
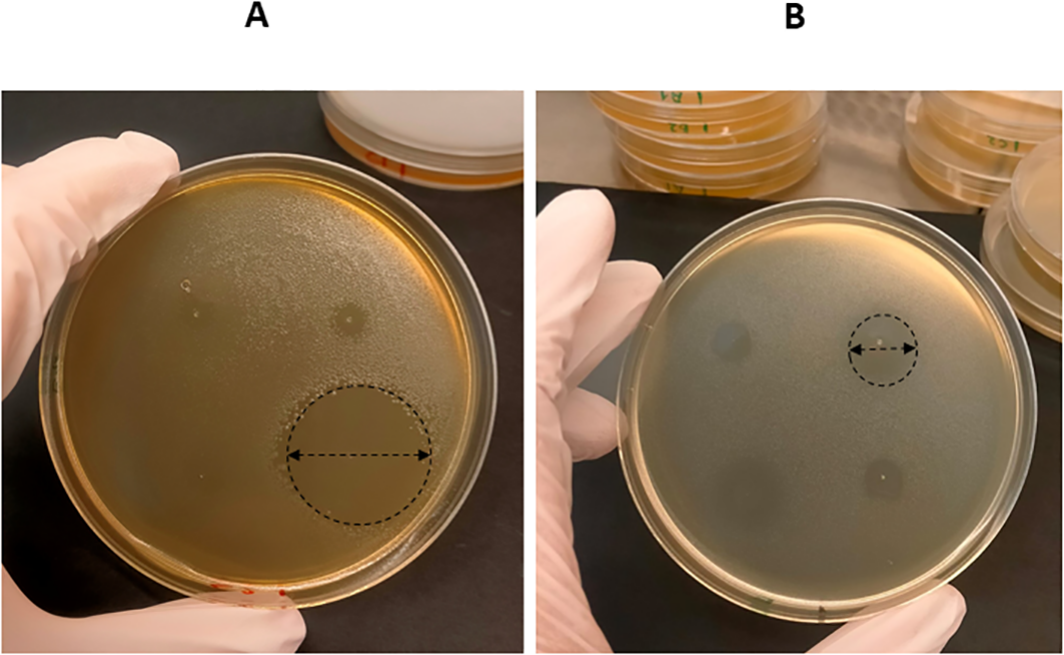
The spot-on-agar test. The diameters of haloes generated by the droplets (one microliter) of essential oils (EOs) spotted onto the agar surface of B98 and B64 plates, which were previously seeded with *P. larvae***(A)** and *M. plutonius***(B)**, respectively, were measured accurately.

As showing in **Figure 2**, the diameters of haloes generated by each EO against *P. larvae* on B98 (A) and *M. plutonius* on B64 (B) agar plates were accurately measured. The mean diameters of the inhibition halos (hds) and SDs were subsequently calculated, as presented in **Table 2**.

**Table 2:** Mean values of inhibition halo diameters of EOs against *P. larvae* and *M. plutonius*.

EO	*P. larvae* mean hd ± SD	*M. plutonius* mean hd ± SD	*p-*value
Cinnamon	1.00 ± 0.21	1.83 ± 0.62	* *p* = 0.033
Lemon	0.78 ± 0.13	0.0 ± 0.0	
Cumin	1.48 ± 0.43	0.0 ± 0.0	**** *p* < 0.0001
Juniper	0.92 ± 0.10	2.00 ± 0.45	** *p* = 0.0014
Lavender	0.80 ± 0.24	0.78 ± 0.26	
Tea tree	0.73 ± 0.08	0.88 ± 0.13	
Peppermint	0.94 ± 0.24	0.95 ± 0.18	
Basil	0.54 ± 0.15	0.77 ± 0.32	
Oregano	3.00 ± 0.0	2.75 ± 0.87	
Black pepper	0.82 ± 0.21	1.20 ± 0.24	
Sage	0.65 ± 0.16	2.83 ± 0.26	**** *p* < 0.0001
Clove	1.08 ± 0.20	0.88 ± 0.62	
Thyme	2.02 ± 0.93	0.88 ± 0.35	*** *p* = 0.0006
Bergamot, Laurel, Myrtle, Rosemary, Ginger	0.0 ± 0.0	0.0 ± 0.0	

The table presents the mean values and standard deviations of the diameters of inhibition halos (hds) in centimetres. The antimicrobial activity of an EO is classified as follows: very high (hd ≥2.0 cm), high (1.5≤ hd <2.0 cm), moderate (1.0≤ hd <1.5 cm), low (0.8 ≤ hd <1.0 cm), and null or very low (hd < 0.8 cm). *p*-values were calculates using two-way ANOVA followed by Sidak’s multiple comparisons test.

The statistical analysis demonstrated a positive interaction (E x P, *p* < 0.0001) between the exposure to EOs (E) and the foulbrood bacterial pathogen (P) under investigation. As shown in **Table 2**, significant differences were identified between *P. larvae* and *M. plutonius* with regard to cinnamon, cumin, juniper, sage and thyme.

Oregano EO exhibited the highest antimicrobial activity against both *P. larvae* and *M. plutonius*, with mean hds of 3.0 and 2.75 cm, respectively. Juniper and sage EOs also exhibited very high antimicrobial activity against *M. plutonius* (hd ≥2 cm), but low (hd = 0.92 cm) or very low (hd = 0.65) activity against *P. larvae*. Thyme EO, on the other hand, was highly effective against *P. larvae* (hd = 2.02 cm) and low effective against *M. plutonius* (hd = 0.88 cm). Cinnamon EO exhibited moderate antimicrobial activity against *P. larvae* (hd = 1.0 cm), but demonstrated high activity against *M. plutonius* (hd = 1.83 cm). In contrast, cumin and clove EOs were mildly effective against *P. larvae* (1≤ hd <1.5 cm) and were ineffective or only slightly effective against *M. plutonius* (hd = 0.88 cm). Black pepper EO was mildly effective against *M. plutonius* (hd = 1.20 cm) and slightly effective against *P. larvae* (hd = 0.82 cm). The EOs that were ineffective against both foulbrood bacteria included bergamot, laurel, myrtle, rosemary and ginger (hd = 0).

The EOs exhibiting very high, high and medium antimicrobial activity against one or both of the foulbrood bacteria – oregano, juniper, sage, thyme, cinnamon, cumin, clove and black pepper –were investigated for toxicity in adult honey bees.

### 3.2 Antimicrobial activity of EOs against foulbrood bacteria: broth microdilution tests

**Figure 3:**
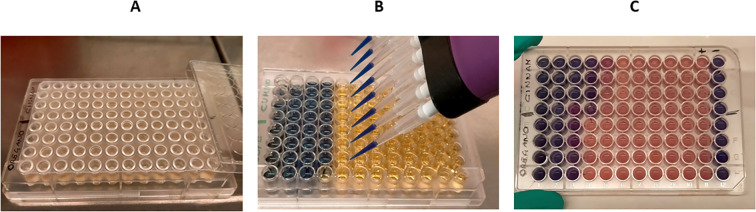
The broth microdilution method based on resozurin. **(A)**, each EO was investigated against *P. larvae* or *M. plutonius* in quadruplicate using 96-well plates. EVA push caps were used to prevent EO evaporation and contamination. **(B)**, following the incubation period (four days for *P. larvae* and seven days for *M. plutonius*), a 0.01% resozurin solution was added to each well. The plates were then incubated for a further 24 hours. **(C)** The plates were evaluated for colour changes. MIC values were defined as the lowest concentration that prevented the growth of the tested foulbrood pathogen. This was determined by observing the absence (blue) or presence (pink) of bacterial growth in the samples. +, growth control containing no EO; ₋, sterile control without bacteria.

The most effective EO on spot-on-agar tests that were oregano, thyme, cumin, clove and cinnamon for *P. larvae* and sage, oregano, juniper, cinnamon and black pepper for *M. plutonius* were further investigated. The microdilution method based on resazurin was employed as a colorimetric assay to determine the MIC values, as shown in **Figure 3**.

The most effective EOs against *P. larvae* were oregano, cumin, clove and cinnamon with MIC values of 2.5 μL/mL, followed by thyme with an MIC value of 5.0 μL/mL. The most effective EOs against *M. plutonius* were cinnamon (MIC = 1.25 μL/mL), followed by oregano (MIC = 2.5 μL/mL), juniper and black pepper (MIC = 5.0 μL/mL), and sage (MIC = 10.0 μL/mL).

### 3.3 Toxicity effects of EOs to honey bee workers

**Figure 4:**
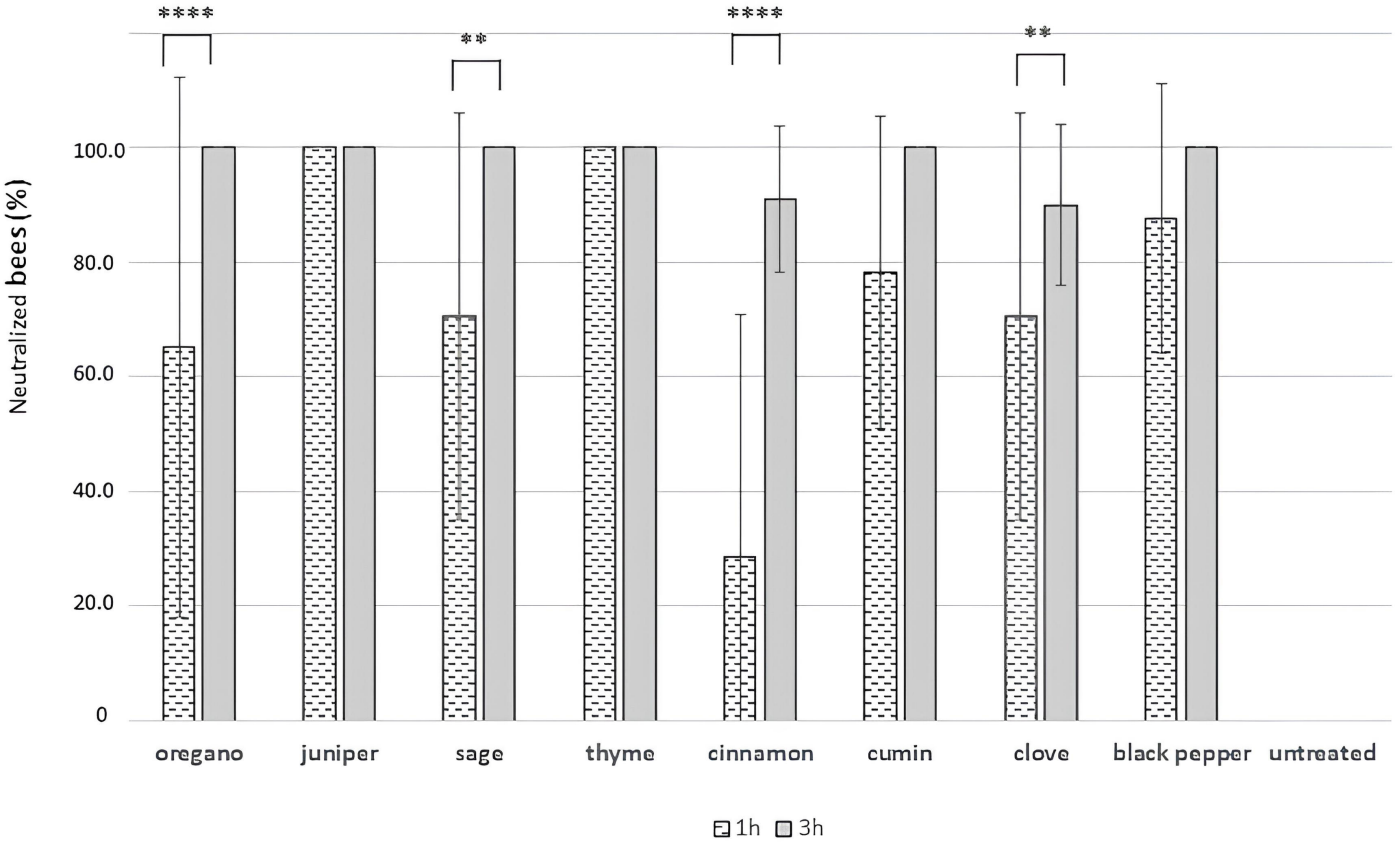
The weighted mean neutralisation rates and their respective standard deviations for both the treated and untreated groups are shown after one and three hours of exposure. Between-group analysis by two-way ANOVA followed by Sidak’s multiple comparisons tests, were performed to determine the significance of the main effects of EO exposure and exposure time, as well as of the interaction between them. Asterisks indicate statistical significance: **p ≤ 0.01 and ****p ≤ 0.0001.

The results of the exposure bioassay to oregano, juniper, sage, thyme, cinnamon, cumin, clove and black pepper EOs, following one hour and three hours of exposure to a concentration of 50 µL/L air, as well as that of the unexposed control, are presented in **Figure 4**.

Both the EO exposure (E, *p <* 0.0001) and exposure time (T, *p <* 0.0001) affected the mean neutralisation rates and their interaction (E x T, *p <* 0.0001) was significant. Significant differences were found between one and three hours of exposure for oregano, sage, cinnamon and clove (**Figure 4**).

Following one hour of exposure to EO vapours, an average neutralisation rate of 100% was recorded for juniper and thyme. The least toxic vapours were those of cinnamon, followed by oregano, sage and clove with mean neutralisation rates of 28.57% (SD = 42.43), 65.22% (SD = 47.16), 70.59% (SD = 35.36) and 70.59% (SD = 35.36), respectively.

The toxicity of the EOs increased drastically in relation to the time of exposure (1 *versus* 3 hours of exposure). Following a three-hour exposure to EO vapours, the majority of EOs demonstrated the capacity to neutralise the entirety of the exposed bees, with the exception of clove and cinnamon, which exhibited mean neutralisation rates of 88.24% (SD = 14.14) and 90.48% (SD = 12.73) respectively. Following a 20-hour exposure period, all exposed bees were entirely neutralised, with the exception of the control group.

## 4 Discussion

The present study assessed the efficacy of 18 EOs against the American and European foulbrood pathogens *P. larvae* and *M. plutonius*, and tested their safety in honey bees.

Essential oils and their monoterpenes are widely studied as an alternative strategy for controlling pests and diseases in honey bees (22, 36–38). It has been demonstrated that EOs can affect the integrity of bacterial membranes. In particular, the hydrophobic components of EOs have been shown to disrupt the structures of the cell wall and cytoplasmic membrane, as evidenced by the increased permeability observed in *P. larvae* (22). Furthermore, the ability to disrupt membranes has been found to correlate with the composition of volatile compounds in EOs (22). *M. plutonius* has been investigated less extensively, and the mechanism by which EOs act against this bacterium has not yet been elucidated. However, given that it is also a Gram-positive bacterium, it is reasonable to hypothesise that analogous mechanisms may take place.

In accordance with a previous study in which the disk diffusion method was used (21), oregano and thyme were found to be the most effective EOs against *P. larvae* by spot-on-agar test. Conversely, oregano exhibited equivalent efficacy to cumin, clove and cinnamon in combating *P. larvae* when the broth microdilution method was employed, with thyme being the subsequent agent of choice. The most effective EOs against *M. plutonius* as determined by spot-on-agar test were sage, oregano and juniper, while clove and thyme demonstrated low efficacy and were outperformed by cinnamon. However, a previous study found that clove demonstrated superior antimicrobial activity against *M. plutonius* than cinnamon when tested by the disk diffusion method (24). The application of the microdilution method revealed that among the tested EOs, cinnamon exhibited the most notable efficacy against *M. plutonius*, followed by oregano, juniper, and black pepper. Conversely, sage proved to be the least efficacious of the tested EOs. Despite the fact that broth microdilution remains the most reliable method for establishing the true potency of an EO (27, 40), the outcomes of MIC determinations are primarily contingent on the solubility of the examined EO. The poor dispersal of water-insoluble compounds of an EO in a liquid growth medium may provide a rationale for the observed discrepancy between the results obtained in our study by the two testing methods. However, irrespective of the method employed, oregano and cinnamon were confirmed to be the most efficacious EOs against both the tested foulbrood pathogens. Furthermore, cinnamon was found to be more toxic to *M. plutonius* than *P. larvae* in both methods. Ceccotti et al. (25) found that the EOs extracted from *C. zeylanicum* bark and leaves were more effective against *P. larvae* strains than *M. plutonius* strains when tested by the agar diffusion test using wells filled with the tested EO. Laurel, myrtle, basil and rosemary were found to be ineffective or only slightly effective against *P. larvae* and *M. plutonius*. However, the EOs of basil and rosemary were found to be effective against *P. larvae* in a study conducted by Kacainiova et al. (21) where disk diffusion test was used. Flesar et al. (39) found that myrtle EO exhibited antimicrobial activity against *P. larvae* and was superior to those of laurel and rosemary in combating AFB when tested by the broth microdilution test. The observed discrepancy between our results and those reported in the extant literature may be attributed to the use of divergent testing methods, types of growth media, solvents and/or emulsifiers for dissolving an EO in water and inoculum density. It has been reported that these factors have the capacity to affect the magnitude of the antimicrobial activity exhibited by EOs (27, 40). Currently, there is no standardised methodology for assessing the antimicrobial properties of natural products such as EOs. Both agar and broth diffusion methods have been used, but the most effective method remains to be determined (27, 40). In our study, we used the spot-on-agar test on B98 or B64 agar plates to test *P. larvae* and *M. plutonius*, respectively. The studies mentioned above used the disc diffusion method on Mueller–Hinton medium (21), the agar well diffusion method on MYPGP medium (Mueller–Hinton broth, yeast extract, glucose, dipotassium phosphate and sodium pyruvate) (25), or the broth microdilution method on MYPGP medium (39). The methodology employed for testing, as well as the selection of the medium, can influence the solubility, diffusion range, evaporation and resulting outcomes of the tested EO (40). Therefore, the antimicrobial activities of the EOs reported through *in vitro* testing should be considered preliminary indicators of potency, and further studies will be required to evaluate their performance under real-world apiary conditions.

The discrepancy in the results may also be attributable to the strain-specific characteristics. As demonstrated in the study by González and Marioli (13), various strains of *P. larvae* exhibited disparate levels of growth inhibition when exposed to the same EO, ranging from high inhibition to no inhibition. Differences in susceptibility were also documented by Gende et al. (20), who tested the cinnamon EO against six *P. larvae* isolates from diverse geographical locations in Argentina. Similarly, Ceccotti et al. (25) examined the effects of bistort and cinnamon EOs on three distinct strains of *P. larvae* and *M. plutonius*, documenting variations in susceptibility responses within the same species. The limited number of foulbrood strains that were examined in this study precluded the possibility of determining strain-specific responses to EOs.

It is evident that also the quality of an EO significantly influences its content and composition, and therefore its antimicrobial properties. A thorough characterisation of the EOs utilised in this study via GC-MS measurements has not been undertaken. Nevertheless, reference was made to the manufacturer’s reports and relevant literature for the identification of the primary bioactive components. Due to the lack of direct GC-MS analysis, the present findings should be viewed as a screening of the EOs’ overall efficacy. Future studies involving detailed chemical profiling of each batch will be necessary to definitively correlate specific molecules with the observed bioactivity. Despite the complexity of the chemical composition of an EO, comprising a mixture of low-molecular-weight compounds, the antimicrobial effect is attributable to only a small number of active ingredients. The EO derived through the steam distillation of dried leaves of *L. nobilis* has been demonstrated to be less effective against *P. larvae* strains than the hydroalcoholic extract from laurel leaves (16, 17). Indeed, the extraction method has been found to affect the concentration of certain phenolic compounds, such as flavonoids. These compounds are capable of inhibiting peptidoglycan synthesis (41), damaging microbial membrane structure (42), modifying bacterial membrane hydrophobicity (43) and modulating quorum sensing (44). In summary, the extraction method employed exerts a significant influence on the antimicrobial properties of an EO. However, steam distillation is the most common industrial method of producing EOs.

In addition to the antimicrobial properties of EOs against the foulbrood pathogens, the toxicity effects of EOs to honey bees have been investigated in order to identify the EOs that were unable to compromise the survival of bees. In this study, worker bees of mixed ages were used to better simulate the heterogeneous population of a colony. While age-matched cohorts are often preferred to minimize biological noise, the assessment of a diverse range of worker ages allows for a more representative evaluation of how a potential apiary treatment might affect the various functional roles (e.g., nurses and foragers) simultaneously present on the frames during an outbreak. Several laboratory experiments have been conducted to assess the toxic effects of EOs by fumigation (vapour-only exposure), complete exposure (contact and fumigation) and spraying towards bees (14). Nevertheless, the fumigation method has been identified as the optimal delivery approach for EOs in both laboratory and field tests (45). In this study, we investigated the toxic effects of EOs exhibiting high or moderate antimicrobial activity against one or both of the foulbrood bacteria in adult honey bees at a concentration of 50 µL/L air. The concentration of 50 µL/L air was selected as a standardized challenge dose based on a review of LC_50_ values in literature (36, 46), adjusted to account for the high-saturation environment of our sealed-bottle vapor bioassay. The EOs of oregano, juniper, sage and thyme, as well as cinnamon, cumin, black pepper and clove, were among the substances analysed.

Following a one-hour exposure period, cinnamon was found to be the least toxic EO to bees, with oregano, and sage and clove ranking second and third, respectively. In contrast, Lindberg et al. (46) discovered that cinnamon was more toxic to clove when bees were completely exposed (contact and fumigation) to the clove EO at concentrations ranging from 18 to 450 μL/L. In a separate study, clove was found to be more toxic to bees than oregano when a single dose of EO ranging from 0.1 to 150 μL was applied to the dorsal surface of a bee worker (47). In a similar manner, clove demonstrated superior toxicity effects in comparison to oregano, thyme, cinnamon and peppermint when bees were completely exposed to 37 μL/L of EO for a period of four hours (36). However, when the duration of exposure was extended to 24 hours, the toxicity of oregano and thyme was found to be superior (36). The most toxic EOs were identified as thyme and juniper, which neutralised 100% of bees after one hour of exposure. The complete neutralisation of the bees was also achieved for oregano, sage and clove after three hours of exposure. Damiani et al. (48) found that thyme was lethal to bees when tested at concentrations ranging from 7-57 μL/L using the complete-exposure method, with the lethality of thyme increasing over time (from 24 to 72 hours of exposure). In consideration of all the above, it appears that both the dosage and the time frame of exposure have the capacity to influence outcomes to a greater extent than the method of exposure utilised for EO testing. However, it is important to note that the present study focused on adult bees. Since AFB and EFB primarily target the larval stage, the lack of toxicity and efficacy data on larvae represents a limitation. Although the EOs showed potential in our current model, further *in vitro* larval bioassays are strictly necessary to confirm their safety before considering apiary applications.

In conclusion, the present study has identified oregano, juniper, sage, thyme, cinnamon, cumin, clove and black pepper as the most effective EOs against the two strains of *P. larvae* and *M. plutonius* that were tested by the spot-on-agar test. The vapors from these EOs have been observed to exert toxic effects on adult bees, albeit with varying degrees of intensity, with cinnamon EO being the less toxic. The use of EOs in beekeeping to contrast AFB and EFB represents a promising field of research. Nevertheless, the development of innovative EO-based formulations requires the conduction of field studies to substantiate findings in such challenging environmental settings, particularly in relation to the type of EO, dose, time, and method of administration to bees.

## Data Availability

The original contributions presented in the study are included in the article/supplementary material. Further inquiries can be directed to the corresponding author.
